# Musculoskeletal tumors and tumor-like lesions with “dark” signal intensity on T2-weighted MR images: A pictorial review

**DOI:** 10.1097/MD.0000000000045179

**Published:** 2025-10-10

**Authors:** Jingkun Zhang, Fengyuan Luo, Juan Chen, Huijuan Yang, Qi Zhang

**Affiliations:** a Department of Medical Imaging, The Affiliated Hospital of Jiangxi University of Chinese Medicine, Nanchang, Jiangxi Province, China; b Department of Radiology, The Second Affiliated Hospital of Nanchang University, Nanchang, Jiangxi Province, China.

**Keywords:** like lesions, low, musculoskeletal tumors, signal T2, tumor, weighted MRI

## Abstract

Musculoskeletal tumors and tumor-like lesions have an indeterminate appearance on magnetic resonance (MR) images and often display varying degrees and extent of T2 hyperintensity. However, a subset of neoplasms and tumor-like lesions may exhibit prominent areas of T2 hypointensity relative to skeletal muscle. The hypointensity observed on T2-weighted MRI can be caused by various substances, including hemoglobin degradation products, lesions containing calcium and calcification substances, mature collagen fibers, melanin, turbulent and rapid blood flow, and highly cellular lesions. Careful evaluation of the presence and pattern of T2 hypointensity in lesions and consideration of potential causes in their associated clinical contexts can help to narrow the differential diagnosis between neoplastic and nonneoplastic lesions. Appropriate interpretation of signal intensity and analyses of lesion location and clinical symptoms enable the correct choice of a further diagnostic algorithm or, in many cases, a final diagnosis determined exclusively by MRI examination.

## 1. Background

Bone and soft tissue tumors and verrucous lesions have multiple classifications and origins, and the imaging manifestations of these presentations are complex, complicating the ability to obtain a definitive preoperative diagnosis by imaging alone relative to the diagnosis of other systemic diseases. Despite these diagnostic difficulties, several diseases have characteristics that largely assist in diagnostic imaging and differential diagnoses, and sometimes, the diagnosis is confirmed by imaging. Most musculoskeletal tumors and tumor-like lesions produce high signals due to high water content, while T2 low-signal lesions are rare. This topic combines clinical and pathology and retrospectively analyzes 6 categories of limited bone and soft tissue tumors and tumor-like lesions that can be manifested as T2WI “dark” low signals, and explains their generation mechanism and imaging diagnosis (Table 1).

**Table 1:** **T2WI “dark” low**-**signal substances and mechanisms.**

Substance	Generation mechanism	Common lesions
Hemoglobin degradation products	Deoxyhemoglobin and intracellular high-iron hemoglobin have strong paramagnetic properties, which significantly shortens T2 time. It contains iron hemoglobin and does not contain H protons, and does not produce signals	Cytacytic repair granuloma, bone giant cell tumor, brown tumor, tendon sheath giant cell tumor, hemophilia, hematoma
Calcium-containing structures	Calcium lacks H protons and does not produce signals	Self-healing/repairing tumors, tumor-like calcium deposition osseous bone tumors, ossifying myositis, crystal deposition disease
Fibers containing lesions	Macromolecular collagen, the movement frequency of hydrogen-bound protons is similar to that of Larmor, and the T2 relaxation speed is extremely fast	Poor fibrostructure, non-ossified fibroma, ligament-like fibroma disease, tendon sheath fibroma, fibrosarcoma
Melanin	Free radicals contain unpaired electrons and have strong paramagnetic properties	Melanoma
Turbulent and rapid blood flow	The turbulent liquid quickly loses phase consistency and the signal is missing	Aneurysm, vascular malformation
Highly cellular lesions	The cell content is high, the arrangement is crowded, the nucleoplasm ratio is large, and the free water content is low	Lymphoma, plasma cell tumor, leiomyoma

## 2. Introduction

With its radiation-free, multiparameter imaging and functional evaluation capabilities, MRI has become the core tool for the diagnosis and treatment of bone and soft tissue tumors, running through the whole process of diagnosis, staging, treatment and follow-up. The soft tissue contrast ability can clearly distinguish between tumor and surrounding muscles, fat, blood vessels and nerves, especially better than CT in showing tumor boundaries, internal structure and surrounding edema. T1 weighted images can show the anatomy and evaluate the fat composition. T2-weighted images and fat suppression sequences can identify the histological composition of tumors.^[1]^ In bone and soft tissue tumors, magnetic resonance imaging usually shows T2 high signals of different degrees and ranges.^[2]^ However, some tumors and tumor-like lesions may show T2 “dark” low signal areas relative to skeletal muscle protrusion. Evaluating the existence and pattern of T2 “dark” low-signal in lesions, speculating on the histological composition of the lesions and considering possible causes in the relevant clinical context are conducive to narrowing the scope of differential diagnosis of the disease, determining the biological behavior of tumors, and guiding the surgical plan.^[3–5]^

## 3. Hemoglobin degradation products

The 3 periods in which hemoglobin shortens the T2 during degradation are acute, early subacute, and chronic.^[6]^ Bone and soft tissue tumors and neoplastic lesions with hemorrhages are common, and when T2WI scans are timed to coincide with the acute, early subacute, or chronic phases of hemorrhage, the images appear “dark” with low signal intensity (Table 2). Some of the more common lesions are giant cell reparative granulomas (GCRGs), giant cell tumors (GCTs) of the bone, brown tumors, diffuse/limited GCTs of the tendon sheath, hemophilia, simple hematomas.

**Table 2:** Hemoglobin breakdown products according to the hemorrhage phase and their T1 and T2 signals.

Hemoglobin breakdown products	Hemorrhage phase	T1 signal intensity	T2 signal intensity
Oxyhemoglobin	Hyperacute	Low	High
Deoxyhemoglobin	Acute	Low/iso	Low
Intracellular methemoglobin	Early subacute	High	Low
Extracellular methemoglobin	Late subacute	High	High
Hemosiderin	Chronic	Low	Low

### 3.1. Giant cell reparative granuloma (GCRG)

**Figure 1.**
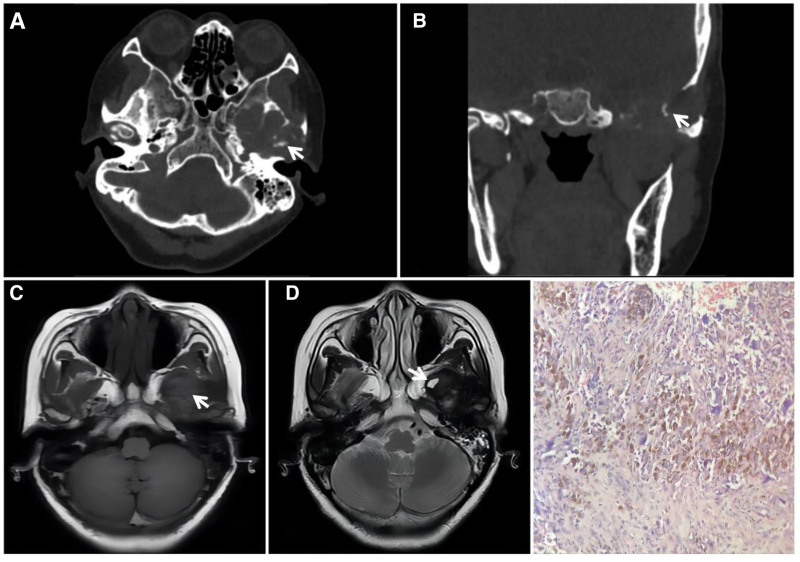
TMJ GCRG, female patient, 54 years of age with a 2-year history of posttraumatic pain and discomfort in the left temporomandibular joint (TMJ) that was recently complicated by limited mouth opening. CT (A, B) showing expansive bony destruction of the left temporomandibular joint fossa, with strips of dense bone-like shadows in the destruction area (↑); T1WI (C) showing localized inhomogeneous flocculent high signals in the lesion (↑); T2WI (D) showing diffuse inhomogeneous “dark” signals throughout the lesion, with small areas of high signal cystic degeneration at the anterior-inferior margins (↑); and histopathological microscopy (E) revealing a large number of multinucleated giant cells of varying sizes and areas of hemorrhage. CT = computed tomography, GCRG = giant cell reparative granuloma, TMJ = temporomandibular joint.

GCRG is a relatively rare nonneoplastic lesion with locally aggressive behavior and predominantly expansive bone destruction in the involved bones. The pathogenesis of GCRG remains unclear and may be related to trauma and intraosseous hemorrhage, typically in the maxillofacial bone, with no obvious sex or age tendencies. The histopathological features of GCRG are as follows: microscopically, GCRG consists of proliferating fibroblasts, areas of metaplastic bone, well-aligned bone trabeculae, areas of vasodilatation containing large numbers of ferritin-containing deposits, and a diffuse inflammatory cellular infiltrate.^[7–9]^ Imaging manifestations include hemorrhage, cystic degeneration, necrosis, and fibrosis within the lesion, and CT reveals uneven density in the soft tissue mass, accompanied by expansive destruction of the adjacent bone. A dense shadow within the lesion is often considered to indicate an osteoid or neoplastic bone, and it has been documented that the longer the onset time is, the more pronounced the bone destruction will be, and the clearer the osteoid and neoplastic bone shadow will be.^[10]^ MR-typical GCRGs exhibit a “dark” low signal on T2W images due to the large number of macrophages phagocytizing ferritin-containing cells in the lesion and a high signal on T2W images when combined with cystic degeneration and necrosis (Fig. 1).

**Figure 2.**
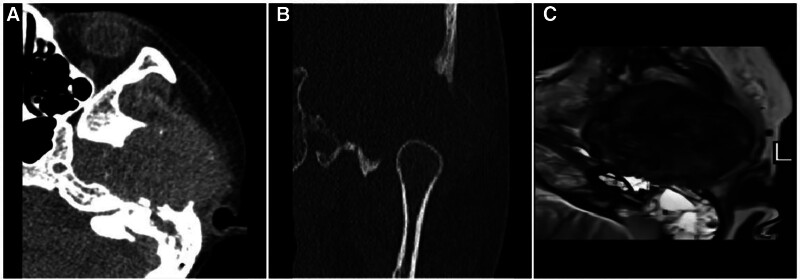
A GCT of the temporomandibular joint fossa from a 29-year-old male with left temporal swelling, pain, and limited mouth opening for 1 year. CT (A, B) showing expansive bony destruction of the left temporomandibular joint fossa with internal soft tissue density and no osteoid or neoplastic bone; T2WI (C) showing diffuse inhomogeneous “dark” signals throughout the lesion, and histochemical H3F3AG34W (+) staining. CT = computed tomography, GCT = giant cell tumor.

Differential diagnosis: differentiating between GCRG and GCT of the bone is important. GCRG is usually cured by complete surgical resection of the lesion, whereas GCT has a high recurrence rate and can become malignant. Notably, GCTs and GCRGs occurring in the maxillofacial bone largely overlap in terms of age of onset and histologic morphology, with similar imaging and gross pathologic manifestations, complicating the preoperative differential diagnosis. GCRGs are generally more bone-like than GCTs, with negative results for the GCRG histochemical H3.3 p.G34W antibody and H3F3A gene^[7,11]^ (Fig. 2).

### 3.2. Giant cell tumor (GCT)

**Figure 3.**
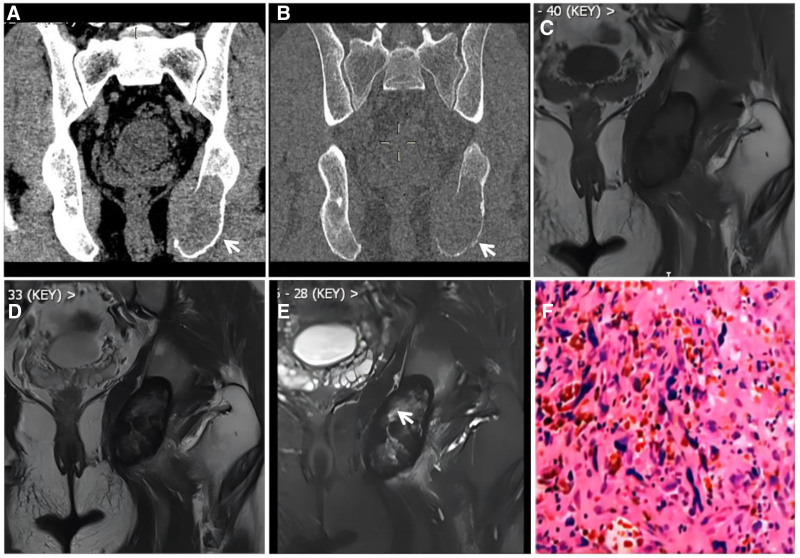
Sciatic node GCT in a male patient, 43 years of age, with left hip pain and discomfort for 6 months that worsened in the sitting position. CT (A, B) showing expansive bony destruction of the left sciatic tuberosity with a visible incomplete bony envelope (↑), no mineralization of the matrix, and no sclerotic margins or periosteal reaction. T1WI (C) showing a heterogeneous low signal, with flocculent areas of lower “dark” signals at the edges and inside the lesion. T2WI (D) showing flocculent “dark” signal areas at the margins and inside the lesion, and FST2WI (E) showing mixed signals, with flocculent “dark” signal areas at the margins and inside the lesion interspersed with irregular punctate high signal shadows (↑). Histopathological microscopy (F) showing large areas of brownish ferritin-containing deposits interspersed with clusters of neoplastic mononuclear cells of varying sizes and reactive multinucleated giant cells. , CT = computed tomography, GCT = giant cell tumor.

GCT is a bone tumor rich in osteoclast-like giant cells. This tumor occurs at the age of 20 to 40 years (after skeletal maturity, rarely before maturity), demonstrates a mild female preference, and typically occurs at the bony ends of the long bones, the anterior part of the vertebral body, and the flat bones and other bone prominences. On histopathological microscopy, GCT demonstrates sheets of tumorigenic mononuclear cells and uniformly distributed giant nontumorigenic multinucleated giant cells, and the interstitium of the tumor contains numerous thin-walled capillaries, which are brittle and prone to hemorrhage. The tumors demonstrate positive results with the H3.3 p.G3 antibody and express the H3F3A gene.^[11,12]^ On imaging, a typical GCT is located at the bony end of the expansive bone destruction site and may have a complete or incomplete shell. The lesion often reaches the adjacent bony articular surface, causing internal hemorrhage and cystic degeneration, and necrosis is significant, resulting in an extremely inhomogeneous lesion signal. In addition, when a large hemorrhage and ferritin deposition occur in the tumor, the T2W image predominantly demonstrates a “dark” low signal (Fig. 3) unless the tumor is combined with pathological fracture, undergoes spontaneous regression, or is subjected to pharmacological treatment; otherwise, there is no periosteal reaction or sclerotic rim.

**Figure 4.**
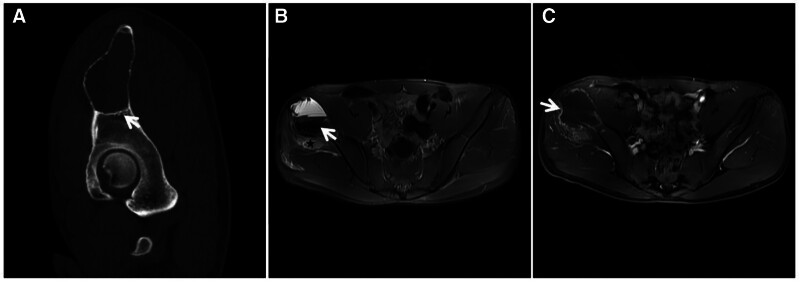
ABC of the ilium in a 21-year-old male with significant pain in the right ilium for 3 days after minor trauma. CT long-axis reconstruction (A) showing expansive bony destruction of the right ilium with well-defined borders and a thin sclerotic rim visible at the medial junction with the normal medullary cavity (↑). FST2WI (B) showing an internal gravity-dependent area of patchy “dark” low signal (↑) with fluid-blood planes and adjacent dorsal soft tissue edema (★) within the lesion, which may have been caused by a microfracture; an enhanced scan (C) showing fine linear enhancement (↑) at the edge of the lesion. ABC = aneurysmal bone cyst, CT = computed tomography.

Differential diagnosis: differentiating GCTs from aneurysmal bone cysts (ABCs with “dark” low signal T2W images) is difficult because they have similar ages of onset, locations, and imaging manifestations. Compared with GCTs, ABCs have more pronounced fluid-blood planes and sclerotic margins, and mature periosteal reactions can occasionally be observed. Due to the sparse parenchymal component of the tumor, the margins and internal septations of the enhancement scans show beehive-like enhancement (Figs. 4 and 6). When imaging differential diagnosis is difficult, histochemistry can be performed first. H3.3 p.G34W positive expression supports the diagnosis of GCT of the bone. If negative, molecular testing can be conducted for H3F3A mutations, the presence of which supports the diagnosis of GCT of the bone.^[11,12]^

**Figure 5.**
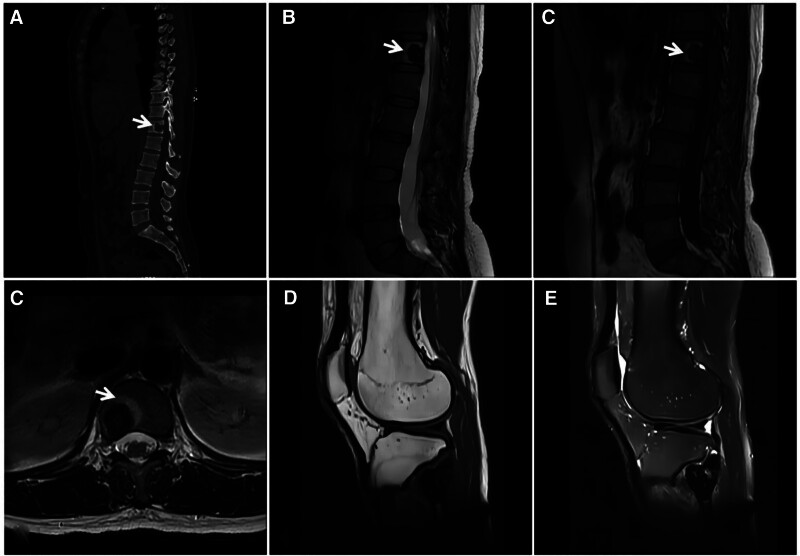
A 44-year-old female diagnosed with primary hyperparathyroidism with lower back and left knee pain for 5 months. CT (A) showing osteolytic bone destruction of the T12 vertebral body with a clear sclerotic margin visible at the edge (↑); MRI (B–F) showing multiple instances of bone destruction of the T12 vertebral body and fibular head, with a diffuse “dark” low signal on T1WI (C, E) and T2WI (B, D, F). CT = computed tomography, MRI = magnetic resonance imaging.

### 3.3. Brown tumor (BT)

Brown tumor manifests as a limited metabolic bone disease caused by hyperparathyroidism, with large deposits of ferritin within the gross pathology of the lesion and a brownish appearance, hence its name. Because hyperparathyroidism is better understood than other etiologies, the skeletal lesions caused by hyperparathyroidism rarely require a puncture biopsy, and a diagnosis can be made definitively by checking blood calcium, phosphorus, and parathyroid hormone levels. On imaging, diagnosis is relatively easy due to the deposition of a large amount of ferritin within the lesion, the diffuse “dark” low signal on T2WI, and the spatial distribution of the lesion, which is characterized by multilocularity, multifocality, and multibony distribution (Fig. 5).^[13]^

### 3.4. Aneurysmal bone cyst (ABC)

**Figure 6.**
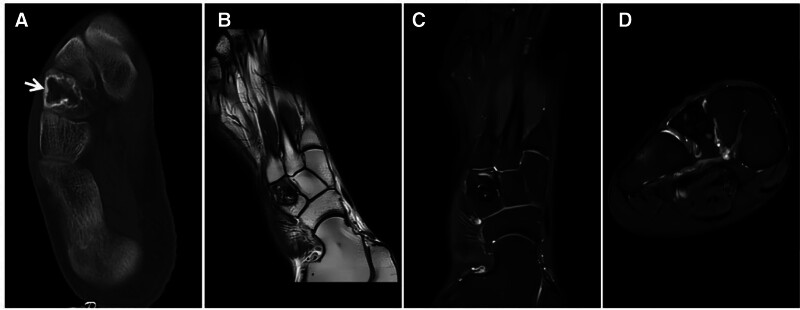
Lateral cuneiform ABC in a male patient, 19 years of age, with right dorsal foot pain and discomfort for 3 years. CT (A) showing a wreath-like sclerotic rim visible at the edge of the lesion (↑); T1WI (B) showing a low signal predominantly mixed-signal image of the lesion; and FST2WI (C, D) showing a “dark” low signal with a predominantly mixed-signal shadow. ABC = aneurysmal bone cyst, CT = computed tomography.

Recent cytogenetic and molecular genetic studies have identified breaks, translocations, and rearrangements of the USP6 fusion gene in most primary ABC lesions,^[14]^ which are classified in the 2020 WHO classification of bone tumors as bone tumors rich in osteoblast-like giant cells. ABC is prevalent between the ages of 5 and 30 years but is most common before the age of 20 years (during periods of active skeletal growth and development), with no significant sex difference observed. The favored sites are the metaphysis of the long bones and the posterior part of the vertebrae. Regarding histopathology, the main feature of ABCs is a spongy cavity with abnormal vascularity and a thin fibrous septum; this cavity is filled with a large volume of blood and ferrous hemosiderin,^[15]^ resulting in a “dark” low signal on T2WI. Typically, the image shows expansive bone destruction, with “honeycomb”-like fibrous segments of varying thickness in the destruction area, multiple planes of fluid and blood, a complete sclerotic rim, a mature periosteal reaction in some of the lesions, and honeycomb-like enhancement of the rims and segments on enhancement scans (Fig. 6).

**Figure 7.**
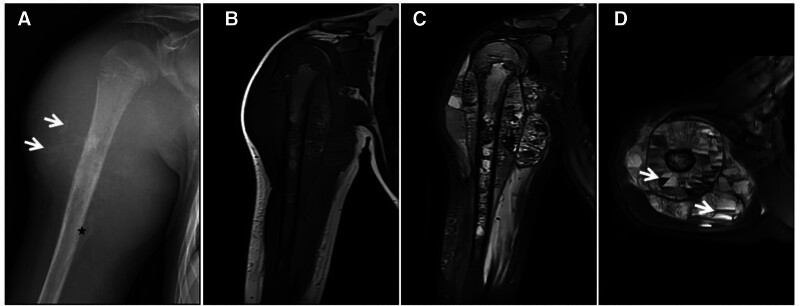
Capillary dilatation osteosarcoma of the humerus. The 16-year-old patient was a male with painful swelling of the right humerus for 6 months, which had recently rapidly worsened. X-ray (A) showing infiltrative bone destruction with a large soft tissue mass in the upper right humerus with irregular verrucae internally (↑) and Codman’s periosteal triangle at the inner and lower margins (★); T1WI (B) showing mixed high and low signals at different times in the medullary cavity and soft tissue mass; and FST2WI (C, D) showing scattered irregular “dark” signal shadows in the medullary cavity and the soft tissue mass, with large planes of fluid and blood (↑).

Differential diagnosis: ABCs are mainly differentiated from GCTs by a “dark” low signal on T2WI, capillary dilatation of osteosarcoma, and other tumors with similar imaging manifestations. Compared with GCTs, ABCs have more pronounced fluid-blood planes and sclerotic margins, with occasional mature periosteal reactions and honeycomb-like enhancement of the margins and internal septa on enhancement scans due to the sparse tumor parenchymal component (Figs. 4 and 6). Compared with that observed in capillary dilatation osteosarcoma, ABC bone destruction tends to be mild, the lesion is well demarcated from the surrounding normal bone, and the rule of periosteal reaction is clear (Fig. 7). When differential diagnosis is difficult, USP6 mutation gene testing can be performed to provide objective auxiliary criteria for the differential diagnosis of the above diseases.^[14]^

### 3.5. Giant cell tumor of the tendon sheath (GCTTS)

**Figure 8.**
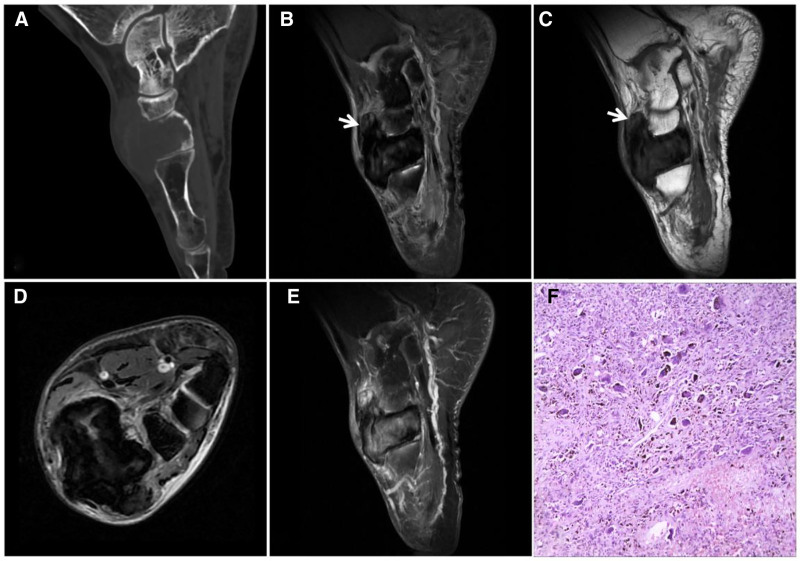
Giant cell tumor of the tendon sheath of the tarsus of the foot. This 47-year-old male patient presented with a giant cell tumor of the tendon sheath of the medial cuneiform bone of the left foot. CT (A) showing expansive bony destruction of the medial cuneiform; T1WI (C) and FST2WI (B, D) showing extensive “dark” signals at the margins and inside the lesion, which locally protrude beyond the bone contour and creep along the synovium (↑); enhanced scan (E) showing markedly inhomogeneous enhancement; and histopathological microscopy (F) revealing a large number of giant cells of varying sizes and phagocytosis of ferroportin-containing cells. CT = computed tomography.

**Figure 9.**
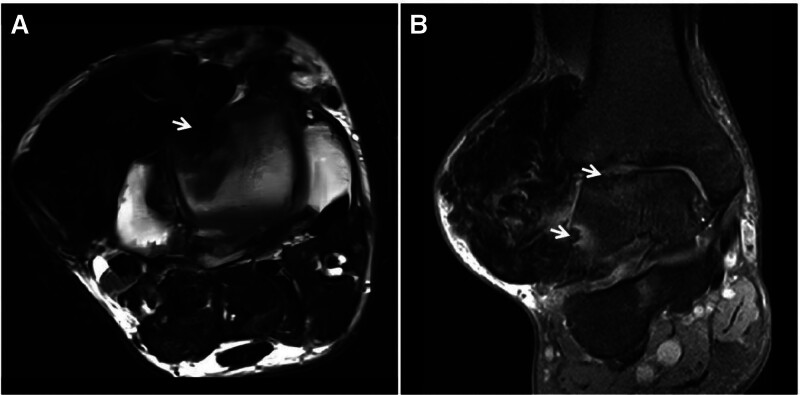
Synovial tendon sheath giant cell tumor in a 30-year-old male with a hard mass in the right outer ankle for 3 years. The tumor slowly increased in size. T2WI (A, B) showing heterogeneous “dark” low signal masses in the lateral and posterior ankles, with long axes parallel to the synovial alignment, accompanied by adjacent bone erosion (↑).

Giant cell tumor of the tendon sheath (GCTTS) is a fibrous histiocytic benign tumor originating in the synovium of the joint. GCTTSs are classified as limited or diffuse according to the lesion’s morphology, growth pattern, and biological behavior. This tumor is more common in young adult females. The limited type is found in the small joints of the hands and feet, whereas the diffuse type is found in the major load-bearing joints, with invasive growth and the potential for malignant transformation.^[16]^ GCTTS originating within the bone are rare and may be associated with the development of adjacent synovial tissue that enters the medullary cavity after a fracture or direct invasion of the bone by the lesion. GCTTSs are rich in capillaries, which are prone to recurrent hemorrhage, leading to the deposition of ferritin and a “dark” low signal on T2WI. Imaging reveals swelling-related destruction of the small bones of the hands and feet near the joints, often breaking through the interosseous compartment and progressing to the outside of the bone, with “dark” low signal predominantly inhomogeneous mixed signals on T2WI and significant enhancement on enhancement scans^[17]^ (Fig. 8). Some T2W images demonstrate a very low signal invasive mass originating from the synovium, with the long axis parallel to the synovium (Fig. 9).

Differential diagnosis: GCTTSs must be distinguished from GCTs and ABCs in the small bones of the hands and feet that demonstrate “dark” low signals on T2W images. Unlike GCTs, GCTTSs often tend to creep along the synovium when they break through the interosseous compartment toward the extraosseous bone, and cystic degeneration and necrosis are rare inside the lesion. Unlike ABCs, GCTTSs exhibit invasive growth that is rich in capillaries, often lacks obvious sclerotic rims, and demonstrates significant enhancement on enhanced scans. GCTTSs occurring in the synovium are difficult to differentiate from tendon sheath fibromas (see Section 4.4).

### 3.6. Hemophilic arthritis (HA)

**Figure 10.**
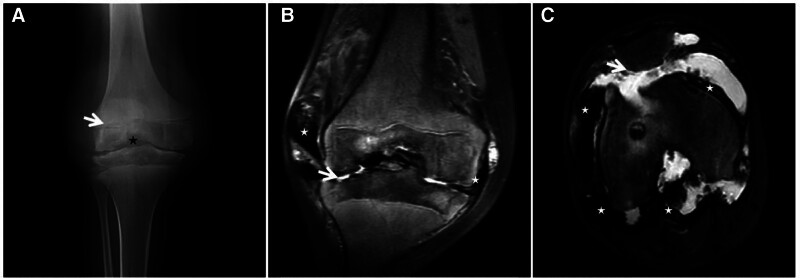
Male, 8-year-old, hemophilia A patient with short stature and pain in both hips, knees, and ankles since childhood, with recurrent unrelieved episodes. X-ray (A) showing uniform narrowing of the right knee joint space, narrowing of the growth plate (↑), and widening and deepening of the femoral ankle fossa (★). The width of the transverse diameter of the distal femur results in a square shape, and T2WI (B, C) shows destruction and disappearance of articular cartilage (↑), effusion in the joint cavity, and diffuse inhomogeneous thickening of the synovial membrane with a “dark” low signal (★).

**Figure 11.**
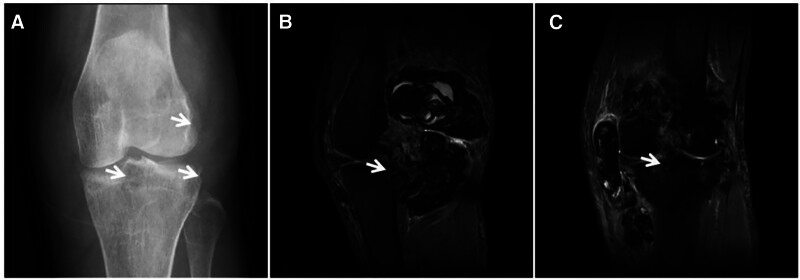
Synovial PVNS in a 52-year-old female patient with left knee pain and dyskinesia for 2 years. X-ray (A) showing swelling of the left knee with bony erosion of the joint margin (↑) and preserved joint space; FST2WI (B, C) showing irregular nodular and mass-like “dark” low signal growth along the synovial membrane and bursa of the knee joint, with bone erosion at the joint margins and relative preservation of the joint space and articular cartilage (↑). PVNS = pigmented villous nodular synovitis.

Hemophilic arthritis (HA) is a recessive inherited bleeding disorder caused by a mutation in an X chromosome gene that results in a deficiency of the associated coagulation factor. HA is a polyarticular disease characterized by irreversible damage to joints due to prolonged and recurrent intra-articular hemorrhage. Men are affected in childhood, and women are carriers. HA is most common in the knee, hip, ankle, and major joints, and the knee joint is the most susceptible.^[18,19]^ HA is now well studied and can be diagnosed by a positive family history, a history of recurrent spontaneous joint bleeding or bleeding after minor trauma, and laboratory tests for prolonged activated partial thromboplastin time and factor VIII or IX deficiency. Due to long-term repeated synovial hemorrhages, inflammation, and massive deposition of ferritin-containing hemoflavin, MR images typically reveal diffuse villous thickening of the synovial membrane and a low T2WI signal. Nutritional disorders of the articular cartilage, uniform narrowing of the articular space, and destruction of the cartilage in the central part of the joints widen and deepen the ankle fossa of the femur and enlarge the ankle fossa. The growth of the bone stops prematurely, with the longitudinal diameter becoming shorter and the transverse diameter becoming longer, causing the bone to appear square in shape^[19]^ (Fig. 10). The differential diagnosis must consider pigmented villous nodular synovitis, which is characterized by a “dark” low signal on T2WI. Unlike HA, pigmented villous nodular synovitis commonly occurs in adults, involves a single joint, and rarely has a polyarticular onset, with a predominance of marginal bone erosion, rare involvement of the intercondylar fossa, and relative preservation of the joint space (Fig. 11).

### 3.7. Hematoma

**Figure 12.**
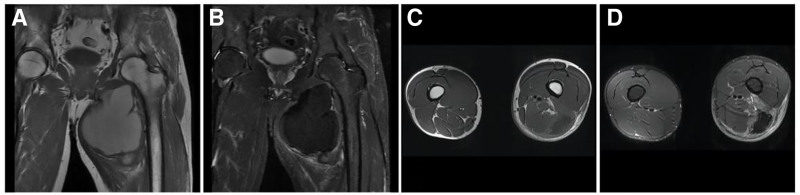
Demonstration of 2 traumatic hematomas at different sites and during different periods; subacute early hemorrhage of the left vastus lateralis group with high tension. T1WI high signal (A); FST2WI (B) “dark” low signal. The lesion was significantly reduced after 1 month; acute phase hemorrhage in the biceps femoris of the left thigh, T1WI low signal (C); T2WI “dark” low signal (D), and edema of the surrounding soft tissues.

As previously described, when the T2WI scan is conducted in the acute, early subacute, or chronic phase of hemorrhage, a “dark” low signal is observed, and such presentations are often characterized by a history of trauma or hemorrhagic disease, with an acute onset and rapid changes in the short term; thus, the diagnosis is typically easy (Fig. 12).

### 3.8. Hemangioma

**Figure 13.**
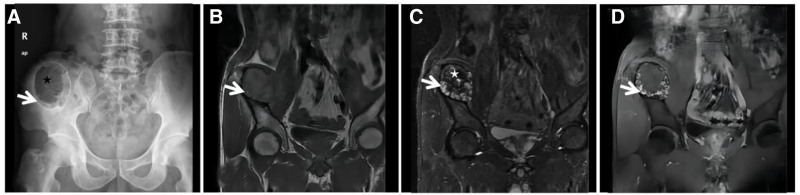
Flat bone hemangioma in a 52-year-old male patient with soreness and discomfort in the right lumbar region for more than 10 years. X-ray (A) showing expansive bony destruction of the right iliac wing with reactive proliferative corded bone trabeculae (★) with clear borders and sclerotic margins visible (↑); T1WI (B) showing a patchy, slightly high signal hemorrhagic area in the interior and margins of the lesion (↑); FST2WI (C) showing a “dark,” inhomogeneous low signal in the center of the lesion (☆), with a petal-like high signal at the margins (↑); and enhancement scan (D) showing significant enhancement of the margins in an irregular manner (↑).

In past studies, hemangiomas were considered vascular malformations in nature and tumor-like lesions. According to the 2020 WHO Classification of Bone and Soft Tissue Tumors, hemangiomas are considered benign tumors comprising tumor-like proliferating vascular tissue. Hemangioma can occur at any age, with a predominance in middle-aged people and a clear female preference. The sites of occurrence are most often the vertebrae, flat bones, and soft tissues. The tumor grows slowly and has a good prognosis. On pathohistology, the tumor interior is honeycombed with sclerotic bone trabeculae and scattered blood cavities and sinuses containing varying quantities of hemorrhagic areas, fat, smooth muscle, fibrous tissue, and thrombi. On imaging, hemangiomas occurring in the vertebral body exhibit the typical polka-dot sign^[20]^; hemangiomas occurring in long bones and flat bones often exhibit well-defined expansive bone destruction, with reactive hyperplasia of cord-like trabeculae, which may stimulate the surrounding periosteum, causing it to proliferate, and the margins of the arterial phase of the enhancement scan exhibit marked lace-like characteristics.^[21–23]^ Due to the large number of spongy sinuses within the tumor body, the T2WI signal intensity is often high, and sometimes, when bleeding is dominant within the tumor body, the T2WI signal is dark and low (Fig. 13).

Differential diagnosis: hemangiomas occurring in the vertebral body and soft tissues have more characteristic imaging manifestations than other lesions. Such manifestations include the polka-dot sign, vein stones, margins of significant enhancement, and centripetal-like enhancement features. Hemangioma diagnosis by imaging is relatively easy. Hemangiomas presenting with “dark” low signals on T2W images are sometimes difficult to distinguish from ABCs. Compared with hemangiomas, ABCs have more fluid and blood planes with fibrous internal septa and rare reactive hyperplastic bone trabeculae, and enhancement scans show honeycomb enhancement of the internal septa and margins. When differential diagnosis is difficult, USP6 mutation gene testing can be performed to provide objective secondary criteria.^[14]^

## 4. Calcium-containing structures

Calcium-containing structures (cortical bone and calcifications) are typically hypointense on T1W and T2W images. Calcium has immobile protons bound to macromolecules, resulting in signal voids most apparent in T2-weighted MR images.^[2,24]^ Common lesions include self-healing, reparative, tumor-like calcium deposits, osteogenic bone tumors, ossifying myositis, and crystal deposition disease.

### 4.1. Period of self-healing and lesion repair

**Figure 14.**
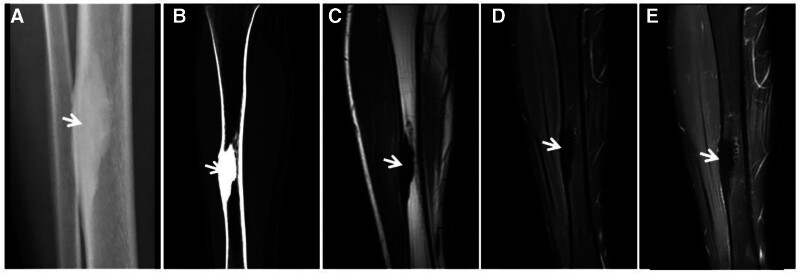
A 20-year-old male diagnosed with “right tibia NOF” 10 years ago without any treatment was recently reviewed after trauma. X-ray (A) and CT (B) images showing an area of eccentric sclerosis (↑) in the medullary cavity of the mid-tibia fused to the adjacent bone cortex with clear borders; T1WI (C) and FST2WI (D) showing a homogeneous “dark” low signal (↑), and enhancement scan (E) showing no enhancement (↑). NOF = nonossifying fibroma.

Some bone tumors exhibit self-limiting effects on growth and development. As time progresses, tumor growth can stop, spontaneous apoptosis can occur, and the tumor can degrade; additionally, the body can repair, remodel, and dystrophically proliferate or even ossify. Additionally, lesions on T2W images exhibit a “dark” low signal or even no signal during these processes. These self-healing characteristics are seen in benign tumors and a small portion of malignant tumors after radiotherapy; theoretically, all tumors or neoplastic lesions regress and are repaired if there is a large area of calcification or ossification, which can manifest as a “dark” low signal on T2W images (Fig. 14).

### 4.2. Tumoral calcinosis (TC)

**Figure 15.**
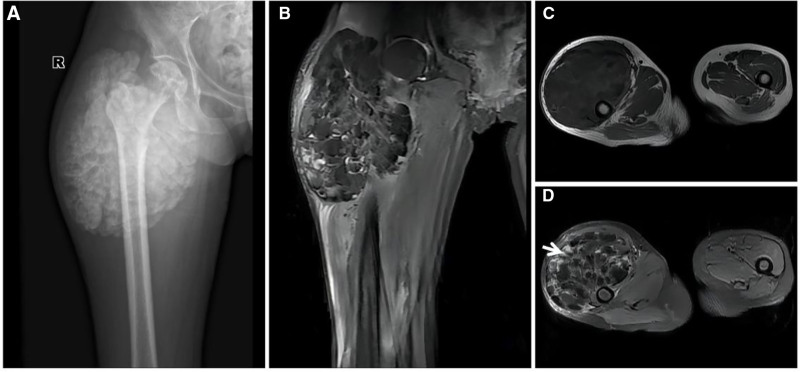
A 44-year-old female patient with stage V CKD secondary to tumor-like calcium deposits around the right hip. X-ray (A) showing an inhomogeneous mulberry-shaped high-density mass with well-defined borders around the right hip joint. T1WI (C) showing a heterogeneous low signal. FST2WI (B, D) showing a heterogeneous “dark” low signal dominated by mixed signals, as indicated by the “sinking sign” (↑). CKD = chronic kidney disease.

Tumoral calcinosis (TC) manifests as soft tissue calcium deposits near large joints and does not involve the synovium. The 3 types of TC are classified according to their etiology: familial: inborn abnormalities of calcium metabolism and autosomal dominant inheritance, with a tendency to occur in families; idiopathic: the cause of the disease is unknown; and secondary: often secondary to diseases involving abnormal calcium and phosphorus metabolism, such as renal failure.^[25]^ The presence of a large number of thin-walled, highly permeable capillaries in the lesion brings abundant calcium ions to the bloodstream, which accumulate locally as a large quantity of crystalline or amorphous calcium, most commonly secondary to uremia. Typically, T2W images reveal mixed-signal masses with a “dark” low signal background in soft tissues near the large joints of the extremities, and sometimes, amorphous liquid calcium planes (sedimentation signs) are observed as characteristic manifestations^[26,27]^ (Fig. 15). Additionally, the diagnosis of TC in combination with patient history is relatively easy, and there is little need for differential diagnosis.

### 4.3. Osteogenic osteosarcoma

**Figure 16.**
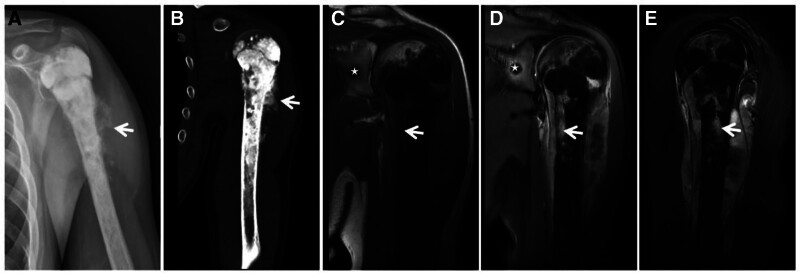
Osteogenic osteosarcoma patient (male, 14 years of age) with pain and swelling in the left shoulder for 6 months that rapidly increased in size for 1 month. X-ray (A) and CT (B) showing extensive osteogenic destruction of the medullary cavity of the upper middle portion of the left humerus, with a large amount of ivory-like tumor bone visible with unclear borders, involving the proximal epiphysis; irregular periosteal osteogenesis is also visible (↑). T1WI (C) and FST2WI (D, E) showing an obvious low signal in the osteogenic area and a T1WI isosignal and T2WI high signal in the nonosteogenic area (↑), surrounded by an irregular mixed-signal soft tissue mass; the scapula and neck can be seen across the joints of the “jumping foci” (★).

**Figure 17.**
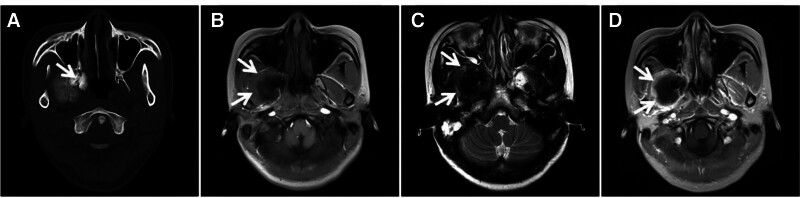
A 28-year-old male patient with pterygoid osteogenic osteosarcoma. CT (A) irregular tumor bone is observed in the right pterygoid with blurred borders and localized ivory-like density (↑); MRI T1WI (B) and T2WI (C) showing a “dark” low signal, with a ring-shaped soft tissue signal mass visible at the edge (↑); enhancement scan (D) showing no enhancement of the “dark” signal area and significant enhancement of the surrounding soft tissue mass (↑).

Osteogenic osteosarcoma is the most common primary malignant bone tumor in adolescents; it predominantly affects the long bones of the limbs and is characterized by the ability of proliferating malignant cells to produce a large quantity of bone-like stroma and tumor bone,^[28]^ which appear as “dark” low signal or no signal on T2WI sequences. Typically, irregular bone destruction occurs in the medullary cavity of the ends of the long bones of the extremities with a soft tissue mass, and the tumor bone can be seen in the medullary cavity and soft tissue mass. The T2W image has a “dark” low signal with a high nonosteogenic signal. It has an area of destruction that is poorly demarcated from normal bone, lacks a sclerotic margin, and is surrounded by swelling of the bone marrow and soft tissues. Interaction between the tumor and adjacent periosteum can produce various forms of periosteal reaction, and occasionally the tumor can grow discontinuously in the medullary cavity or even in a jumping pattern across the joint (Figs. 16 and 17). Overall, osteogenic osteosarcoma imaging is typical, and the diagnosis is easy. The degree and extent of tumor invasion, including paraneoplastic, vascular and neurological involvement, and distant metastases, are used to select a surgical plan. Differential diagnosis is considered unnecessary.

### 4.4. Myositis ossificans

**Figure 18.**
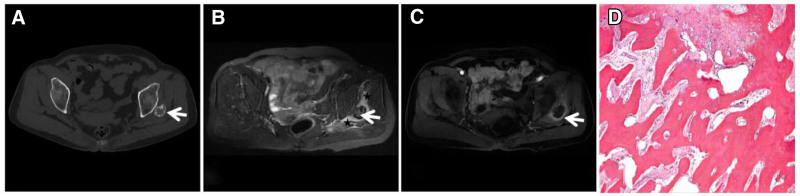
Ossifying myositis in a 69-year-old female patient with posttraumatic pain in the left hip for 35 days. CT (A) showing an eggshell-like hyperdense shadow (↑) around the left hip with well-defined borders and an isointense central area; FST2WI (B) showing a circular “dark” low signal lesion with a slightly high signal in the center (↑) and surrounding soft tissue edema (★); enhancement scan (C) showing circular enhancement (↑); and pathohistological microscopy (D) revealing a large number of mature bone trabeculae with inflammatory cell infiltration. CT = computed tomography.

Myositis ossificans is a benign, nonneoplastic lesion with predominantly reactive new bone hyperplasia that occurs within muscle or other soft tissues. This disease can arise at any age and demonstrates no significant sex differences. Patients often have a history of local trauma or surgery and develop myositis ossificans within a relatively short period of 1 to 2 weeks after the trauma. The most striking histopathological features are the active proliferation of fibroblasts, the loose distribution and disordered arrangement of cells within the lesion, the regular arrangement of fibroblasts in the periphery, the production of reactive new bone by osteoblasts, and the initial eggshell-like ossified area seen in the periphery of the lesion due to the continuous calcification of the osteoid. As the lesion matures, ossification becomes more pronounced, and the lesion progresses from the periphery to the center of the lesion, resulting in the formation of mature trabecular bone structures.^[29–31]^ In terms of imaging manifestations, initial MRI showed unclear boundaries and mixed signals, as well as infiltration and extensive swelling of the surrounding soft tissues; this was initially misdiagnosed as a malignant tumor. As the ossification matures, the lesion’s borders become clearer, and a T2WI “dark” ring of low signal calcification can be seen around it. With time, the ossification of the lesion becomes more obvious, the T2WI “dark” low signal area becomes enlarged, and the lesion size and surrounding soft tissue edema reduce (Fig. 18).

**Figure 19.**
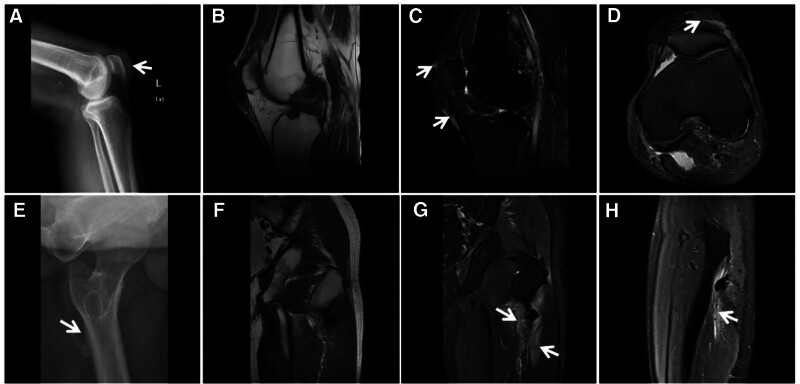
(A–D) Gout, X-ray (A) showing limited swelling and increased density (↑) in the prepatellar subcutaneous soft tissue; T1WI (B) showing a low signal; and T2WI (C, D) showing a “dark” low signal with mild swelling of the surrounding soft tissue (↑). (E–H) Hydroxyapatite deposition disease; X-ray (E) showing striated calcification at the left gluteus maximus stop and reactive hyperplasia of the adjacent periosteum (↑); and T1WI (F) and FST2WI (G, H) showing a homogeneous “dark” low signal with surrounding soft tissue edema (↑).

Differential diagnosis: It is difficult to differentiate the initial stage of ossification from malignant tumors. Complete or incomplete T2W images of the “dark” low signal ring around the lesion are more helpful in diagnosing ossifying myositis. In addition, ossifying myositis often involves a history of local trauma or surgery and occurs within a relatively short period after the trauma. The duration of the disease can provide objective auxiliary criteria for differential diagnosis. In the advanced stages, ossifying myositis must be differentiated from crystal deposition disease. Crystal deposition occurs in the articular cartilage, synovium, and tendons, while osteomyositis occurs in the muscles. Osteomyositis has uneven signals in the ossified area and reflects the transition direction of ossification maturation from the periphery to the center, with clear calcification at the edges and blurring in the center and uneven signals on T2WI, with “dark” low signals at the edges and low signals in the center. Crystal deposition disease shows a homogeneous “dark” low signal on T2WI (Fig. 19).

### 4.5. Crystal deposition disease

Calcium-based crystals are abnormally deposited in articular cartilage, ligaments, the synovium/bursa, and tendons and are common in middle-aged and older adults; additionally, hydroxyapatite deposition disease/calcium pyrophosphate dihydrate deposition disease and urate deposition disease are the most common clinical conditions.^[32,33]^ Imaging reveals focal or diffuse calcific lesions in the cartilage, articular ligaments, synovium/bursa, and tendons, with a “dark” low signal on T2WI and reactive edema and swelling of the surrounding soft tissues in the acute phase, with erosion of adjacent bony structures (Fig. 19). Crystal deposition disease has typical site and imaging features and is easily diagnosed but must be differentiated from ossifying myositis (see Section 4.4).

## 5. Fibers containing lesions

Fibers are an extracellular matrix secreted by connective tissue cells, and their main component is large collagen molecules with little free water and abundant bound water. The motion frequency of water-bound hydrogen protons is similar to that of the Larmor frequency, resulting in extremely fast T1 and T2 relaxation speeds and, thus, a “dark” low signal on T2WI. Similarly, when the tumor consists mainly of a large volume of fibrous tissue, the T2WI signal is significantly reduced. Common lesions include fibrous tissue-rich lesions, such as fibrous dysplasia (FD), nonossifying fibromas (NOFs), ligament-like fibromatosis, and tendon sheath fibromas.

### 5.1. Fibrous dysplasia (FD)

**Figure 20.**
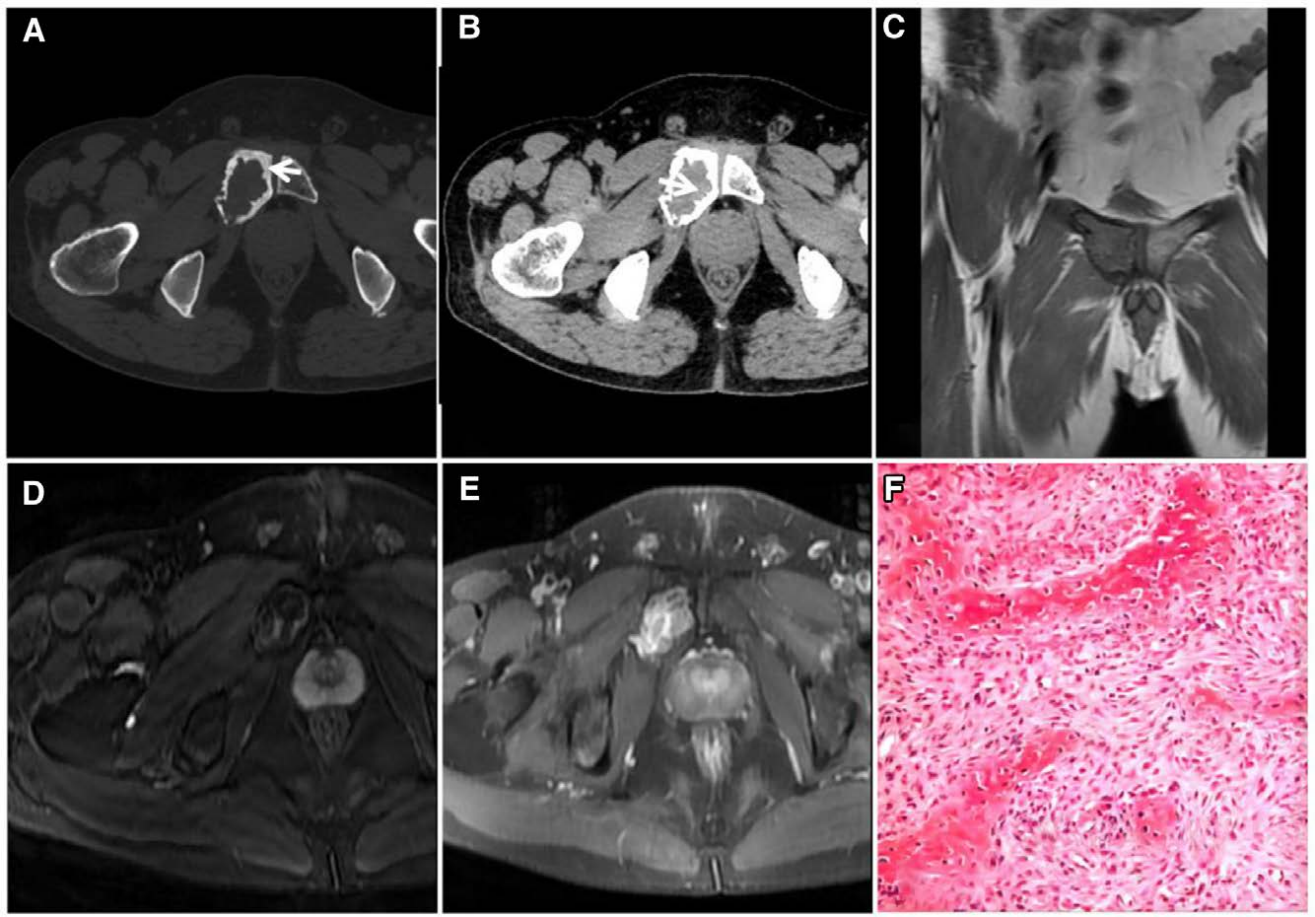
FD in a 46-year-old male patient with motor right pubic bone pain for 10 years. CT (A, B) showing expansive bony destruction of the right pubic bone with thickening and sclerosis of the bone cortex at the margins (↑) and inhomogeneous soft tissue density within (↑), with no matrix mineralization. T1WI (C) showing an iso-low signal. FST2WI (D) showing a predominant “dark” signal. Enhancement scan (E) showing marked inhomogeneous enhancement, and histopathology revealing a large number of fibroblasts and immature bands of woven bone (F). FD = fibrous dysplasia.

Bone dysfunction in FD occurs when bone development stops at the immature woven bone stage, at which point the body fails to form normal trabeculae. FD can occur in any bone, with maxillofacial bones, skulls, ribs, and long bones being the most commonly affected; it can also be observed at any age, with no apparent sex preference. The main histopathological features include fibroblast proliferation, dense collagenous matrix production, immature woven bone formation, and an interstitium with more abundant thin-walled blood vessels, which may be accompanied by metaphyseal bone, foam cell aggregates, a matrix that may undergo markedly mucus-like changes, and islands of hyperdifferentiated cartilage; rarely, mature lamellar bone is observed.^[34]^ The pathology of FD tissue is complex, and on T2WI, the areas of fibrous tissue and mineral deposition are low, while the areas of mucinous changes and cartilage differentiation are high; therefore, when the pathology of FD tissue is dominated by fibrous tissue, it can show a “dark” low signal on T2WI (Fig. 20).

### 5.2. Nonossifying fibroma (NOF)

**Figure 21.**
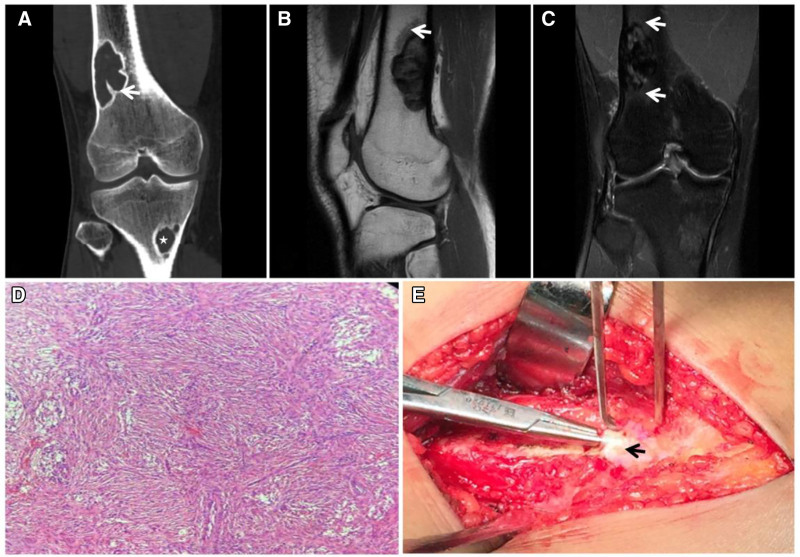
A 18-year-old male patient with NOF with pain in the lower right thigh for 15 days after strenuous exercise. CT (A) showing eccentric bony destruction of the medullary cavity of the right lower femur with well-defined borders and an intact sclerotic rim on the medullary side (↑), and a similar lesion is also observed in the proximal tibia (★). MRI T1WI (B) and FST2WI (C) showing a heterogeneous “dark” signal with slight edema of the adjacent bone marrow (↑); histopathology (D) showing a large number of matted fibroblasts microscopically. Intraoperative image (E) showing white stromal tough tissue (arrow). CT = computed tomography, NOF = nonossifying fibroma.

NOF is a benign, self-limiting spindle cell tumor of the bone containing large numbers of osteoblastic giant cells and storiform structures^[35]^ that occurs at the bony ends of the long tubular bones of the lower extremities. NOF occurs in children and adolescents, mostly at the age of 5 to 20 years, and may stop growing and show self-limitation after the bone matures.^[36]^ Histopathology reveals mainly mat-arranged spindle-forming fibroblasts interspersed with varying proportions of osteoblast-like multinucleated giant cells and foam cells, with no osteogenic tendency. Typical images show intracortical or subcortical expansive, eccentric bone destruction, often with an intact sclerotic rim on the medullary side that is clearly demarcated, with large mineral deposits in the limited matrix. When the histopathologic component is predominantly fibrous, the T2W image shows mixed signals against a background of “dark” low signals (Fig. 21). Notably, imaging is required to confirm a diagnosis of NOF, and coupled with its self-limiting nature, active clinical treatment is generally not administered unless there are obvious symptoms or pathologic fractures.

### 5.3. Desmoid-type fibromatosis (DTF)

**Figure 22.**
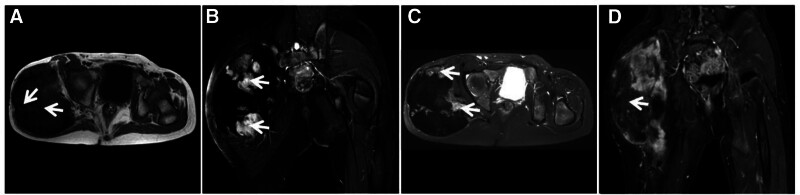
DTF in a 14-year-old female patient with a painless, stiff mass on the right buttock for 4 years that slowly increased in size. T1WI (A) showing a large mixed low signal soft tissue mass in the right buttock with scattered streaks of “dark” signal (↑); FST2WI (B, C) showing a “dark” low signal mass with scattered clusters of high signal (↑) growing in the direction of the gluteus maximus muscle and parallel to it in the long axis. Enhancement scan (D) showing no significant enhancement of the “dark” signal area (↑). DTF = desmoid-type fibromatosis.

Fibroblasts/myofibroblastic tumors are rare and can be divided into 2 types, 1 originating from the soft tissue and 1 from the bone. The soft tissue type mainly originates from muscles, tendon membranes, or deep fascia and is considered deep fibromatosis. Desmoid-type fibromatosis (DTF) is an intermediate tumor with insignificant cellular anisotropy and no distant metastasis that is histologically benign but locally invasive and prone to recurrence. However, DTFs can undergo spontaneous regression and exhibit self-limiting growth.^[37]^ DTF tumors are most common in adults but can also occur in children and adolescents. In addition, DTF tumors have been found in the abdominal wall, upper limb bands, chest wall, back, thighs and forearms, head and neck, intra-abdominal cavity, and mesentery. Microscopically, the tumors consist of fibroblasts, fibroblasts, and collagen fibers in varying proportions, with fibroblasts and fibroblasts interspersed or spirally arranged. The tumors are usually surrounded by abundant collagen bundles exhibiting homogeneous staining and inconspicuous cellular anisotropy.^[38]^ Imaging reveals a single regular or irregularly shaped mass, with the lesion growing along the muscle, tendon membrane, or deep fascia, with the long axis parallel to these regions. The lesion’s border is unclear, and it may infiltrate adjacent structures or cross the intermuscular compartment. The MR signal characteristics are related to the proportion and distribution of the histological components in the lesion. Specifically, the T2WI signal is high in areas with a large number of cellular components and low in areas with a high number of collagen fibers. Necrosis, calcification, and liquefaction are rarely observed. In addition, when there is a large proportion of collagen fibers, the lesion shows a band-like “dark” low signal shadow on T2WI, and there is no significant enhancement, which is the more characteristic manifestation of DTF^[38,39]^ (Fig. 22).

**Figure 23.**
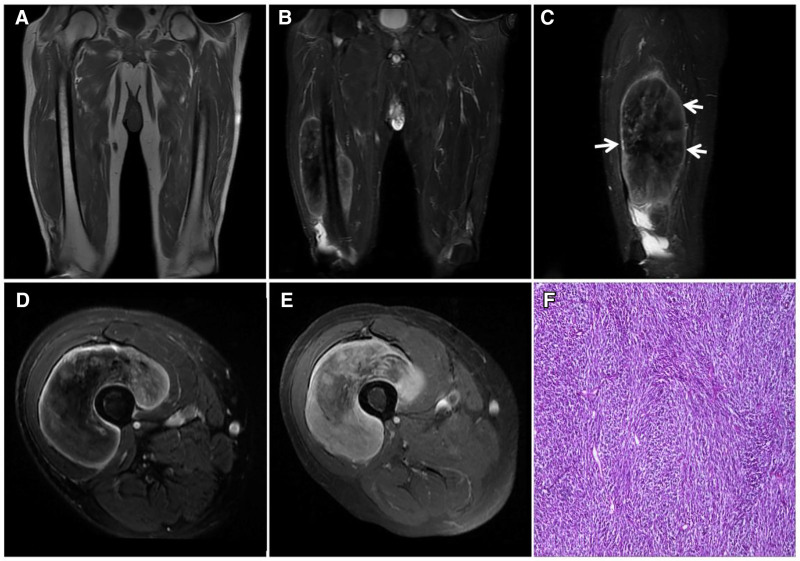
An 86-year-old male with a fibrosarcoma in the right mid-thigh and a mass growing around the femoral stem in the deep anterior interosseous space of the right mid-thigh. T1WI (A) showing a low signal; T2WI (B–D) showing a heterogeneous “dark” low signal with clear borders and a visible high signal pseudoenvelope (↑); enhancement scan (E) showing markedly inhomogeneous enhancement; and pathohistology (F) showing a microscopic view of a large number of tightly packed fibroblasts with large, crowded nuclei accompanied by abnormal karyorrhexis and anomalous cells.

**Figure 24.**
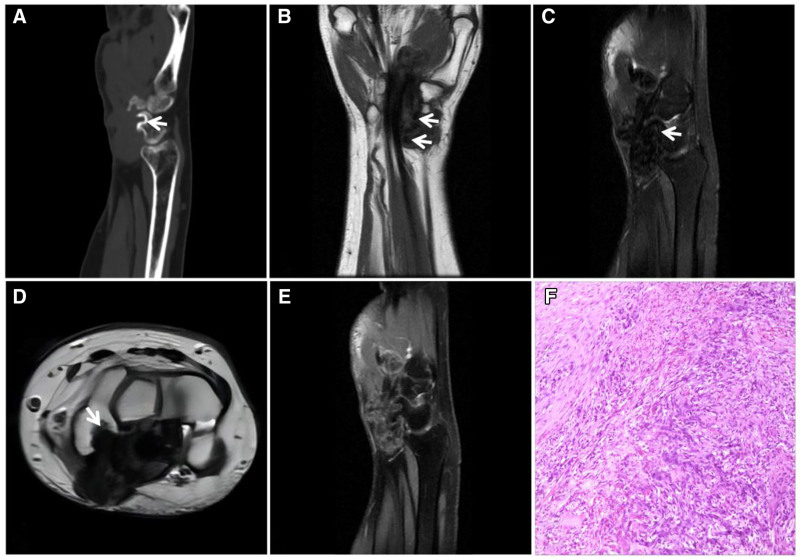
A 67-year-old female patient with carpal tunnel tendon sheath fibroma. CT (A) showing a soft tissue density mass in the carpal tunnel along the flexor tendon with erosion of the adjacent carpal bones and sclerotic margins (↑); T1WI (B) showing a low signal with scattered irregular “dark” signal areas (↑); and T2WI (C, D) showing a “dark” low signal with adjacent carpal erosion (↑). Enhancement scan (E) showing markedly inhomogeneous enhancement. Pathohistology (F) showing a large number of fibroblasts with a disorganized arrangement.

Differential diagnosis: DTF must be differentiated from tenosynovial fibroma (Fig. 24) and highly differentiated fibrosarcoma (Fig. 23); however, it is almost impossible to differentiate them on imaging because they have similar ages of onset, locations, imaging manifestations, and histologic patterns, demonstrating the limitations of imaging and the requirement of pathology for a definitive diagnosis.

### 5.4. Fibroma of the tendon sheath (FTS)

Like DTF, fibroma of the tendon sheath (FTS) is a benign fibroblastic/myofibroblastic tumor that develops in the tendon sheaths of the forearm, with the flexor tendon sheath (palmar side) being the most common site of occurrence. FTS is common in individuals aged 20 to 50 years and more common in men than women. Histopathologically, FTS consists of dense fibrous tissue containing spindle or stellate mesenchymal cells. It has been suggested that at the final stage of development, FTS may be a tenosynovial GCT; thus, FTS and tenosynovial GCTs may constitute different stages of the same disease.^[40]^ Moreover, on imaging and histopathology, tenosynovial GCTs and tenosynovial fibromas have the same manifestations and are indistinguishable (Figs. 9 and 24). Pathologically, tenosynovial fibromas can harbor USP6 gene rearrangements,^[41]^ which may be the only difference between these 2 pathologies.

## 6. Melanin

**Figure 25.**
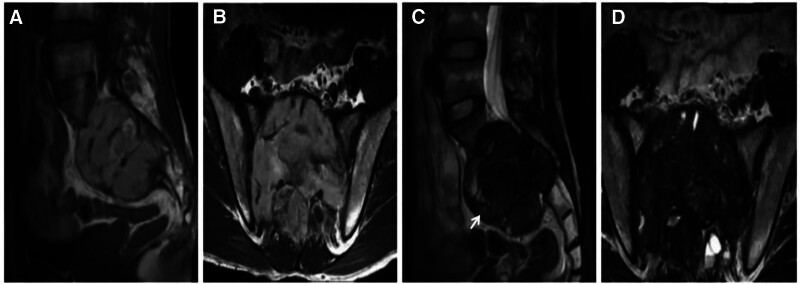
Sacral primary melanoma, TIWI (A, B) has a high signal, T2WI (C, D) has a “dark” low signal, the posterior sacral canal is obviously narrowed, the sacral hole is occluded, and the formation of extraosssal soft tissue mass can be seen (↑).

The free radicals of melanin contain unpaired electrons and have a strong paramagnetic effect, leading to shortened T1 and T2 relaxation times.^[2,42]^ MRI can reveal 4 manifestations according to the melanin content: melanin type: T1WI high signal and T2WI low signal; nonmelanin type: T1WI low signal or isosignal and T2WI high signal or isosignal; mixed type: signals of different heights and lows; and hemorrhagic type: bleeding signals in different periods of MR manifestations. Only when the melanocyte content reaches more than 10% will there be a typical MR performance.^[43]^ Melanoma is a highly malignant melanocytic tumor most commonly found in the skin of the extremities. Melanoma can also be found outside the skin in the mucous membranes, respiratory tract, gastrointestinal tract, heart, reproductive system, choroid, and soft meninges of the eye. Melanoma readily metastasizes and has a high recurrence rate and poor prognosis. This tumor is mostly observed in middle-aged and older adults; the older the age is, the greater the incidence rate is. Melanoma affects more men than women. Previous melanoma studies have focused on the central nervous system. Primary melanoma of the bone is rare, and few studies have been conducted on this presentation; no more than 10 cases have been reported worldwide. MRI-guided pigmented melanoma is characterized by a high signal on T1WI and a “dark” low signal on T2WI^[44]^ (Fig. 25), complicating the diagnosis and differential diagnosis of nonpigmented, mixed, and hemorrhagic melanoma.

## 7. Turbulent and rapid blood flow

**Figure 26.**
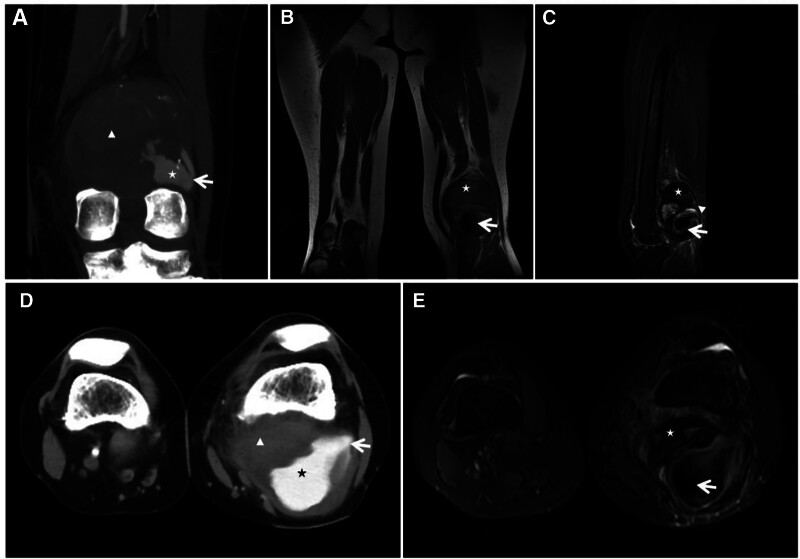
Pseudoaneurysm in a 56-year-old female patient with a palpable pulsatile mass in the popliteal fossa for 1 month that gradually increased in size. CTA (A, D) showing a rupture of the medial wall of the popliteal artery in the left knee (↑), contrast leakage (★), and an encapsulated hematoma around the vessel (▲). MRI T1WI (B) and FST2WI (C, E) showing mixed “dark” signals (↑) demonstrating the flow-void effect produced by high-speed blood flow near the rupture and a decreased blood flow rate around the distal rupture, forming an encapsulated multiperiod hematoma (★) and an intact fibrous peritoneum (▲).

Turbulent liquids can rapidly lose phase coherence, causing signal loss and resulting in no signal on T2WI, an effect known as the “flow-through phenomenon.” This phenomenon can occur within normal blood vessels or in luminal structures with fast-flowing blood.^[45,46]^ The flow-through phenomenon can reveal normal vessels on T2-weighted images and lesions, such as aneurysms, or other less common vascular malformations (Fig. 26).

## 8. Highly cellular lesions

**Figure 27.**
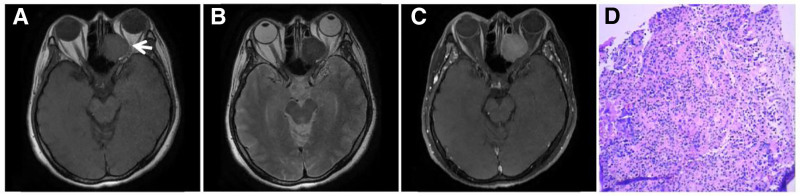
A 51-year-old female patient with diffuse large B-cell lymphoma of the sinuses. T1W (A) showing a homogeneous, slightly high signal mass in the left posterior group of sieve sinuses with clear borders and compression of the left retro-orbital tissues (↑); T2W (B) showing a homogeneous “dark” low signal, (C) homogeneous mild enhancement, and (D) microscopic diffuse small round cells with large, deeply stained, crowded nuclei.

**Figure 28.**
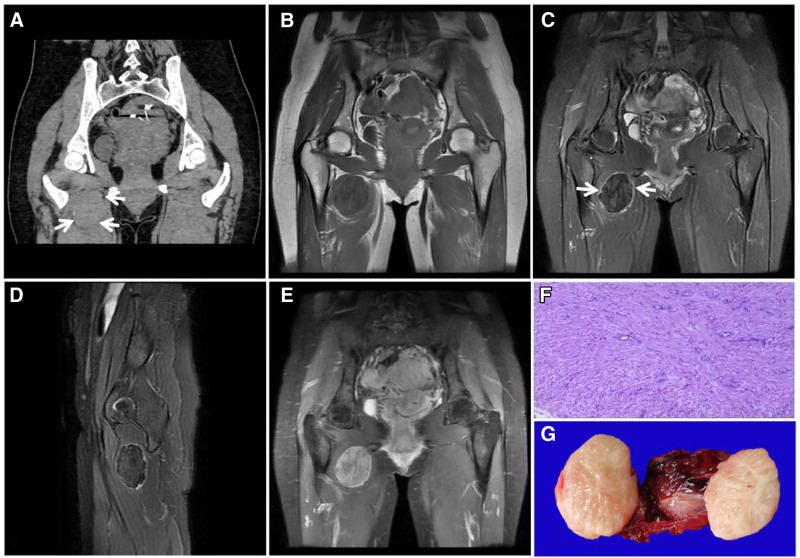
A 41-year-old female patient with a smooth muscle tumor in the interstitial space of the right adductor group. CT (A) showing a classically rounded isointense mass in the interstitial space of the right retractor muscle group with well-defined borders and visible fat surround (↑); MRI T1WI (B) showing an iso-low signal; FST2WI (C, D) showing a “dark” low signal surrounded by a ring-shaped high signal pseudoenvelope (↑); enhanced scan (E) showing marked enhancement of the lesion and periphery; pathohistology (F) showing a microscopic mass of crowded arrangement of smooth muscle cells; and macroscopic specimen (G) tough, yellowish-white, without hemorrhage. CT = computed tomography, MRI = magnetic resonance imaging.

Some tumors have a high cellular content, a high nucleoplasmic ratio, and a low free water content, resulting in a low signal on T2W images^[47]^ (Figs. 27 and 28).

## 9. Conclusions

Musculoskeletal tumors and tumor-like lesions with dark signal intensity on T2W images are caused by the shortening of the T2 transverse relaxation time. This shortening is caused mainly by paramagnetic substances (blood, mineral substances, and melanin), a lack of excited protons (turbulent and rapid flow), the high viscosity of colloid material, or decreased extracellular fluid concentration (highly cellular lesions). Most limited bone and soft tissue tumors and tumor-like lesions with “dark” low signals on T2W images are not biologically malignant. Understanding the location and morphology of pathological changes that lead to characteristic T2 high signals can narrow down the scope of differential diagnosis, determine biological behavior, and even in some cases, the final correct diagnosis can be determined by magnetic resonance imaging alone.

## Author contributions

**Conceptualization:** Jingkun Zhang, Juan Chen, Qi Zhang.

**Formal analysis:** Jingkun Zhang, Fengyuan Luo, Juan Chen.

**Investigation:** Fengyuan Luo, Huijuan Yang, Qi Zhang.

**Resources:** Huijuan Yang.

**Supervision:** Jingkun Zhang, Qi Zhang.

**Writing – original draft:** Jingkun Zhang.

**Writing – review & editing:** Qi Zhang.
